# Reemergence of Plasmodium vivax malaria in the republic of Korea.

**DOI:** 10.3201/eid0402.980219

**Published:** 1998

**Authors:** B H Feighner, S I Pak, W L Novakoski, L L Kelsey, D Strickman

**Affiliations:** US Army, Republic of Korea.

## Abstract

Plasmodium vivax malaria reemerged in the Republic of Korea in 1993. The number of cases has tripled each year since, with more than 1,600 cases reported in 1997. All 27 cases in U.S. troops resolved uneventfully with chloroquine/primaquine therapy. Disease is localized along the western Demilitarized Zone and presents minimal risk to tourists.


### Reemergence of *Plasmodium vivax* Malaria in the Republic of Korea

**Brian H. Feighner, Son Il Pak, William L. Novakoski, Lori L. Kelsey, and Daniel Strickman**
U.S. Army, Republic of Korea

*Plasmodium vivax* malaria reemerged in the Republic of Korea in 1993. The number of cases has tripled each year since, with more than 1,600 cases reported in 1997. All 27 cases in U.S. troops resolved uneventfully with chloroquine/primaquine therapy. Disease is localized along the western Demilitarized Zone and presents minimal risk to tourists.

*Plasmodium vivax* malaria has been endemic on the Korean peninsula for centuries (1). The primary vector is believed to be *Anopheles sinensis*, a zoophilic and facultatively anthropophilic mosquito, which breeds in fresh, sun-exposed water such as that found in rice fields (2). During the Korean War (1950–1953), U.S. troops were exposed to malaria (3) and received weekly chloroquine chemoprophylaxis. This regimen generally succeeded in preventing malarial attacks while U.S. troops were in the Republic of Korea. As the troops returned home, however, U.S. military hospitals were inundated with cases of malaria—at one point, as many as 629 cases per week (3). More than 3,000 cases of malaria were documented in U.S. troops that served during the war. Subsequent U.S. military research led to the current use of primaquine therapy (4). The outbreak of malaria continued on the peninsula after the armistice. In the 1960s and 1970s, the Republic of Korea government combined active and passive case detection with extensive use of pesticides in an ambitious eradication project (1). Indigenous transmission of malaria appeared to cease in the mid-1970s, and in 1979 the Republic of Korea was declared malaria-free by the World Health Organization (5).

In 1993, *P. vivax* malaria reemerged in the Republic of Korea along the western edge of the Demilitarized Zone (DMZ) (6). Since the reemergence, the annual number of cases has increased geometrically, tripling (1995, 1996) or quadrupling (1997) in each of the past 4 years (Table 1). In 1997, the Republic of Korea Army administered antimalarial chemoprophylaxis to approximately 8,000 of its troops at greatest risk (U.S. Army/Republic of Korea Army/Republic of Korea Malaria Symposium, pers. comm.). U.S. troops in Korea were not placed on antimalarial chemoprophylaxis in 1997 after local and U.S. Army experts concluded that personal protective measures and community mosquito control would adequately limit transmission (7).

Cases in 1997 were distributed as expected from June to October, with a peak of 518 cases in August (Table 2). As in past years, more than 80% of the cases were associated with military service along the western DMZ. During 1997, 34 cases of *P. vivax* malaria in U.S. troops were attributed to exposure in the Republic of Korea. This number includes 27 cases in U.S. Forces-Korea troops, one case in a soldier stationed in Okinawa, Japan, who was temporarily assigned to the Republic of Korea in August 1997, and six cases in U.S. troops exposed in the summer of 1996. The U.S. Forces-Korea incidence of *P. vivax* in all malarious areas

Table 1. Annual number of *Plasmodium vivax* malaria cases in U.S. Forces-Korea (USFK), Republic of Korea (ROK) Army, and ROK Civilians, 1993–1997

| Year | USFK | ROK Army | ROK Civilians | Total |
|---|---|---|---|---|
| 1993 | 0 | 1 | 1 | 2 |
| 1994 | 1 | 18 | 20 | 39 |
| 1995 | 1 | 98 | 19 | 118 |
| 1996 | 11 | 285 | 71 | 367 |
| 1997 | 27 | 1,154 | 461 | 1,642 |

Address for correspondence: Brian H. Feighner, HHC, 18th MEDCOM, Unit #15281, Box 763, APO AP 96205-0054 USA; 822-7916-3028; e-mail: ltc_brian_feighner@smtplink.seoul.amedd.army.mil.

Table 2. Monthly distribution of *Plasmodium vivax* malaria cases in the Republic of Korea (ROK), by onset of disease, 1997

| Month | USFK[a] | ROK Army | ROK Civilian | Total |
|---|---|---|---|---|
| Jan | 0 | 0 | 0 | 0 |
| Feb | 0 | 0 | 0 | 0 |
| Mar | 0 | 5 | 2 | 7 |
| Apr | 1 | 10 | 3 | 14 |
| May | 0 | 31 | 4 | 35 |
| Jun | 0 | 145 | 38 | 183 |
| Jul | 2 | 260 | 102 | 364 |
| Aug | 11 | 317 | 190 | 518 |
| Sep | 10 | 320 | 75 | 405 |
| Oct | 3 | 55 | 38 | 96 |
| Nov | 0 | 9 | 9 | 18 |
| Dec | 0 | 2 | NA[b] | 2 |

[a]USFK, U.S. Forces-Korea.
[b]NA, Not available.

between July 1, 1997, and September 30, 1997, was estimated to be 0.5 cases per 1,000 per week. The incidence northwest of the Imjin River was approximately one case per 1,000 per week. Site-specific rates, as well as rates for the Republic of Korea Army, cannot be released because of security concerns; however, the Republic of Korea Army has 50 times more exposed soldiers than does U.S. Forces-Korea.

The epidemic remains focused along the western DMZ. Before 1997, almost all civilian cases were attributed to exposure in areas within 10 km of the DMZ. In 1997, however, dozens of cases occurred among civilians farther than 10 km from the DMZ. The area where U.S. troops are exposed is strictly controlled; tourists are not allowed in this area after dark. Other areas under the Republic of Korea Army control, e.g., Yonchon and Chorwon, are accessible to tourists but are seldom visited. The risk to foreign tourists, therefore, appears to be negligible at this time.

Of the 27 U.S. Forces-Korea cases, 18 were in U.S. soldiers and 9 were in Korean soldier augmentees to the U.S. Army. All cases were in men 19 to 40 years of age (median 21.5 years). With one exception, the cases occurred in junior enlisted soldiers. As in the past 3 years, no cases occurred in African American soldiers, who account for almost 30% of the exposed U.S. troops. This is consistent with the known natural immunity against *P. vivax* in African Americans, most of whom do not have the Duffy antigen on their red blood cells (8). This relative immunity of African Americans was also seen during the Korean War (3). The time from onset of symptoms to diagnosis was 1 to 25 days (median 5 days). Three patients with mild illness were treated as outpatients. All patients quickly recovered after standard chloroquine therapy. All patients were also treated with primaquine.

The Korean strain of *P. vivax* malaria has been well characterized by both Korean and U.S. medical personnel (1,3). This type of malaria is termed “temperate climate” *P. vivax* malaria (3). Transmission is restricted to only a few summer months, when the Korean peninsula is warm enough to support the parasite’s reproductive cycle in the mosquito (1,3). This Korean *P. vivax* malaria is remarkable in that 40% to 50% of cases have no symptoms until 6 to 9 months after infection (3,9). The long incubation period interferes with determining the start of the transmission season. Malaria cases begin to occur in the late spring or early summer, before the weather is warm enough to support malaria transmission. These early season cases, arising from infections acquired the previous fall, blend into and fuel infections transmitted each summer. Another result of this long incubation period, given the extraordinary (>90%) annual turnover in U.S. troops, is the return of infected soldiers to the United States before any symptoms appear.

Malaria in the Republic of Korea is also characterized as epidemic or unstable because conditions usually do not support widespread disease (10). Factors contributing to this instability include the extremely short transmission season and the zoophily of the major vector. Areas with unstable malaria usually have low to very low rates of disease but can occasionally have high rates in response to climatic, livestock, or sociopolitical aberrations (10). The current epidemic may fit this pattern. Heavy rains from 1994 to 1996 led to widespread crop failures in the Democratic Peoples Republic of Korea. As a result, domestic farm animal populations, the preferred source of blood for *A. sinensis*, fell sharply. These factors may have contributed to the geometric increase of disease, but the lack of data makes this explanation speculative.

Most Korean officials perceive the current epidemic to be a Democratic Peoples Republic of Korea public health problem spilling over the DMZ and discount the importance of indigenous transmission in the Republic of Korea. Broad-scale epidemiologic investigation is necessary to confirm indigenous transmission. Although it is not known if the epidemic will continue its geometric increase and geographic expansion in 1998, a decrease in disease without extensive intervention seems unlikely.

### Acknowledgments

The authors thank Colonel Daniel F. Perugini, U.S. Forces-Korea Surgeon, and Colonel Kenneth G. Torrington, Deputy Commander, 18th Medical Command, for their support and critical review of this manuscript.

All equipment, supplies, and author salaries were received from the U.S. Army.

### References

1. Paik YH, Ree HI, Shim JC. Malaria in Korea. Japanese Journal of Experimental Medicine 1988;58:55-66.
2. Ree HI, Hong HK, Paik YH. Study on natural infection of *P. vivax* in *Anopheles sinensis* in Korea. Korean J Parasitol 1967;5:3-4.
3. Hankey DD, Jones R, Coatney GR, Alving AS, Coker WG, Garrison PL, et al. Korean vivax malaria. I. Natural history and response to chloroquine. Am J Trop Med Hyg 1953;2:958-69.
4. Jones R, Jackson LS, DiLorenzo A, Marx RL, Levy BL, Kenny EC, et al. Korean vivax malaria. III. Curative effect and toxicity of primaquine in doses from 10 to 30 mg daily. Am J Trop Med Hyg 1953;2:977-82.
5. World Health Organization. Synopsis of the world malaria situation, 1979. Wkly Epidemiol Rec 1981;56:145-9.
6. Cho SY, Kong Y, Park SM, Lee JS, Lim YA, Chae SL, et al. Two vivax malaria cases detected in Korea. Korean J Parasitol 1994;4:281-4.
7. Craig S, Ockenhouse C, Evans E, Keep L, Hewitson W, Bell C. EPICON final report: malaria in U.S. troops, Republic of Korea. Aberdeen Proving Grounds (MD): U.S. Army Center for Health Promotion and Preventive Medicine; 1996.
8. Miller LH, Mason SJ, Clyde DF, McGinness MH. The resistance factor to *Plasmodium vivax* in blacks. The Duffy-blood-group genotype, FyFy. N Engl J Med 1976;295:302-4.
9. Shute PG, Lupasco G, Branzei P, Maryon M, Constantinescu P, Bruce-Chwatt LJ, et al. A strain of *Plasmodium vivax* characterized by prolonged incubation: the effect of numbers of sporozoites on the length of the prepatent period. Trans R Soc Trop Med Hyg 1977;70:474-81.
10. Gilles HM, Warrell DA. Bruce-Chwatt's essential malariology. 3rd ed. Boston: Edward Arnold; 1993. p. 127-31.
